# Annual Research Review: Childhood maltreatment, latent vulnerability and the shift to preventative psychiatry – the contribution of functional brain imaging

**DOI:** 10.1111/jcpp.12713

**Published:** 2017-03-13

**Authors:** Eamon J. McCrory, Mattia I. Gerin, Essi Viding

**Affiliations:** ^1^ Division of Psychology and Language Sciences University College London London UK; ^2^ Anna Freud National Centre for Children and Families London UK

**Keywords:** Child abuse, maltreatment, mental health, functional magnetic resonance imaging, resilience

## Abstract

**Background:**

Childhood maltreatment is a potent predictor of poor mental health across the life span. We argue that there is a need to improve the understanding of the mechanisms that confer psychiatric vulnerability following maltreatment, if we are to progress from simply treating those with a manifest disorder, to developing effective preventative approaches that can help offset the likelihood that such disorders will emerge in the first place.

**Methods:**

We review extant functional neuroimaging studies of children and adolescents exposed to early neglect and/or maltreatment, including physical, sexual and emotional abuse across four neurocognitive domains: threat processing, reward processing, emotion regulation and executive control. Findings are discussed in the context of ‘latent vulnerability’, where alterations in neurocognitive function are considered to carry adaptive value in early adverse caregiving environments but confer long‐term risk.

**Results:**

Studies on threat processing indicate heightened as well as depressed neural responsiveness in maltreated samples, particularly in the amygdala, thought to reflect threat hypervigilance and avoidance respectively. Studies on reward processing generally report blunted neural response to anticipation and receipt of rewards, particularly in the striatum, patterns associated with depressive symptomatology. Studies on emotion regulation report increased activation of the anterior cingulate cortex (ACC) during active emotion regulation, possibly reflecting greater effortful processing. Finally, studies of executive control report increased dorsal ACC activity during error monitoring and inhibition.

**Conclusions:**

An emerging body of work indicates that altered neurocognitive functioning following maltreatment: (a) is evident even in the absence of overt psychopathology; (b) is consistent with perturbations seen in individuals presenting with psychiatric disorder; (c) can predict future psychiatric symptomatology. These findings suggest that maltreatment leads to neurocognitive alterations that embed latent vulnerability to psychiatric disorder, establishing a compelling case for identifying those children at most risk and developing mechanistically informed models of preventative intervention. Such interventions should aim to offset the likelihood of any future psychiatric disorder.

## Abstract 2

Read the Commentary on this article at doi: 10.1111/jcpp.12726

## Introduction

Childhood maltreatment, including physical, sexual, emotional abuse and neglect, arguably represents the most potent predictor of poor mental health across the life span. Such early adversity substantially increases the risk of a wide range of psychiatric disorders during childhood and adulthood (Green et al., 2010; Koenen & Widom, 2009; Vachon, Krueger, Rogosch, & Cicchetti, 2015; Widom, DuMont, & Czaja, 2007). In contrast to compelling evidence characterizing the long‐term impact of maltreatment there is a striking lack of precision in our mechanistic understanding of how maltreatment alters neurocognitive systems in ways that can embed vulnerability to future mental health problems (McCrory & Viding, 2015). As clinicians we therefore remain remarkably ill‐equipped to either identify *or* help those children who are at most risk of developing mental health problems following maltreatment experience. This gap in our knowledge is not accidental; rather it has resulted from the convergence of a number of disparate factors that have inadvertently conspired to inhibit progress.

First, research over the last two decades in the field of child mental health has been primarily organized around a medical model seeking to investigate presenting psychiatric disorders. While clearly important in its own right, this work has rather eclipsed a focus on those mechanisms associated with the *pathogenesis* of psychiatric disorder across development. The study of the presenting psychiatric disorder has traditionally been underpinned by an assumption that individuals who meet criteria for a given disorder are comparable – a notion which is now widely understood to be incorrect as individuals with the same behavioural symptomatology may differ in relation to aetiology and neurocognitive presentation (i.e. equifinality) (Cicchetti & Rogosch, 1996; Gottesman & Gould, 2003; Luking, Pagliaccio, Luby, & Barch, 2016). In line with this, individuals who present with a psychiatric disorder and also have a history of childhood maltreatment, differ in a number of respects from those without such a history. For example, psychiatric disorders in individuals who have experienced maltreatment are likely to develop earlier, with more severe symptomatology (Hovens et al., 2010) and with an increased risk of comorbidity (Harkness & Wildes, 2002). Moreover, a disorder in an individual who has experienced childhood maltreatment is more likely to be persistent and recurrent and less likely to respond to standard treatment approaches (Agnew‐Blais & Danese, 2016; Hovens et al., 2012; Nanni, Uher, & Danese, 2012). Indeed, it has been suggested that individuals within a diagnostic category who have childhood histories of maltreatment may represent specific ecophenotypes (Teicher & Samson, 2013).

A second challenge faced by researchers has been adequately defining and measuring a complex and multifaceted environmental risk factor such as maltreatment (Danese & McCrory, 2015). We will return to this issue later. This challenge is accentuated by the fact that children who experience maltreatment are typically exposed to more than one form of abuse or neglect (Finkelhor, Ormrod, & Turner, 2007; Higgins & Mccabe, 2001) with individual differences in severity, frequency and age of onset. Such difficulties inherent in operationalizing the core construct of interest (maltreatment exposure) in contrast to the ostensibly ‘clear‐cut’ definitions provided by the psychiatric classification system has contributed to maltreatment research being viewed (intentionally or otherwise) as less amenable to mechanistic or neurocognitive research and of lower priority than research focusing on presenting psychiatric disorders.

Fortunately this position is shifting. We would argue that neuroimaging research is providing an important catalyst for this change. Over the last 5 or 6 years a new body of work has emerged using functional magnetic resonance imaging (fMRI) to investigate alterations in neurocognitive systems following maltreatment exposure: this work represents the substantive focus of the current review. There is also a growing emphasis across psychiatry and other disciplines on the development of a preventative model for mental health (McGorry, 2013) and a greater willingness and openness on the part of researchers and journal editors alike to grasp the complexity inherent in the study of childhood maltreatment. This is reflected in the increasing number of research groups and studies that are systematically investigating the neurocognitive mechanisms associated with maltreatment experience. The studies we consider in this review are consistent in delineating how individuals who have experienced maltreatment, even in the absence of psychiatric disorder, present with changes in brain function across social, emotional and cognitive domains. These changes are strikingly aligned with the neurocognitive signatures documented in adults presenting with common psychiatric disorders. Such findings are creating an impetus towards a preventative psychiatry model in which these neurocognitive changes are not conceptualized as signs of ‘damage’ but rather as indicators of ‘latent vulnerability’. We argue that these indicators can provide important clues regarding the pathogenesis of mental health problems at the mechanistic level – and in turn offer potential targets for future preventative interventions (McCrory & Viding, 2015).

### The theory of latent vulnerability

The theory of latent vulnerability reconceptualizes the link between childhood maltreatment and the associated increased risk of psychiatric disorder across the life span (McCrory & Viding, 2015). According to this theory, maltreatment results in measurable alterations in a number of neurobiological systems that reflect calibration to neglectful and/or abusive early environments. A general principle of the theory is that these changes are often beneficial within the early maladaptive context (i.e. carry adaptive value within that particular setting) thus representing in part a functional response. However, such adaptations are equally thought to incur a longer term cost as they may mean that the individual is poorly optimized to negotiate the demands of other, more normative environments, thus increasing vulnerability to future stressors (McCrory & Viding, 2015). Patterns of adaptation are likely to arise at multiple levels (see Cicchetti, 2016). Recently, for example, we demonstrated that young adults self‐reporting childhood experiences of maltreatment displayed altered patterns of epigenetic modulation in genes implicated in a range of physical and psychiatric disorders (Cecil et al., 2016). Gene ontology analyses indicated that individual maltreatment subtypes showed unique methylation patterns enriched for specific biological processes (e.g. *physical abuse*: stress regulation, fear response, heart rate regulation; *physical neglect*: lipoprotein metabolism, polyamine metabolism, regulation of cholesterol efflux). In addition, a ‘common’ epigenetic signature *across* maltreatment subtypes was also observed, enriched for biological processes related to neural development and organismal growth (Cecil et al., 2016).

**Figure 1:**
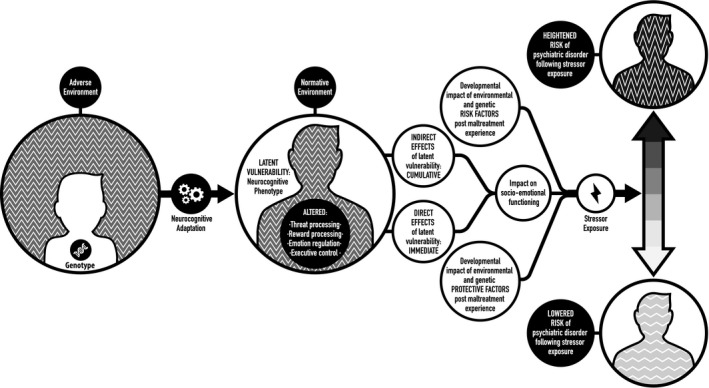
A schematic illustration displaying the embedding of latent vulnerability at the neurocognitive level and differential outcome in relation to psychiatric risk depending on protective factors, stressor exposure and genotypes

However, here, we focus on neurocognitive functioning as the level of investigation most likely to have immediate translational relevance. In this context, we suggest that indicators of latent vulnerability can be thought of as being characterized by three key features:

1. First, these indicators are not necessarily *symptoms* of any future disorder. Rather, they refer to cognitive processes or representations and associated patterns of neural activation that are implicated in the pathogenesis of a disorder. For example, as discussed later in greater length, altered response to reward cues at the neurocognitive level may increase vulnerability to depression (e.g. Dennison et al., 2016; Hanson, Hariri, & Williamson, 2015), but this pattern of altered reward processing does not in itself constitute a symptom of depression.
2. Second, these indicators are best indexed at a *systems level*. In other words, latent vulnerability likely reflects a complex phenotype that can be thought of as a ‘maladaptive calibration’ in higher order systems important for socioemotional and cognitive functioning. Given the heterogeneity of maladaptive (and resilient) outcomes associated with maltreatment across psychiatric disorders (Gilbert, McEwan, Bellew, Mills, & Gale, 2009), it would be reasonable to hypothesize that a limited but varied set of candidate neurocognitive systems are altered in a way that increases or reduces psychiatric vulnerability following maltreatment exposure.
3. Third, these indicators should be present *prior* to onset of psychiatric disorder and help predict level of future risk. That latent vulnerability is present does not necessarily inform us as to the timing of disease onset. Such vulnerability could theoretically be present for months or years, but clinical symptoms may only manifest under certain conditions characterized by stress or developmental challenge, or indeed may never manifest given adequate intrinsic and extrinsic protective factors (e.g. resilient genotypes, social support), and the absence of future stressors, despite the enduring presence of latent vulnerability (see Figure 1). In other words, the emergence of a psychiatric disorder can be understood as the interaction between latent vulnerability and stressor exposure.

Over recent years a series of functional imaging studies have begun to investigate the association between maltreatment and impairments in a number of neurocognitive systems; we suggest that impaired processing in these systems index latent vulnerability and shed light on the mechanisms implicated in the pathogenesis of psychiatric disorder.

## Functional magnetic resonance imaging and the study of childhood maltreatment

In the current review, we focus on the functional neuroimaging literature rather than studies of alterations in brain structure following maltreatment exposure; for those readers interested in the latter we would recommend the excellent review by Teicher and Samson (2016). One advantage of functional studies is that they have the potential to shed greater light on psychological mechanisms, allowing us to investigate with some degree of precision how individuals who have experienced maltreatment process the world differently from their peers. We focus on the studies of children and adolescents only in light of space constraints; however, a consideration of functional imaging studies of adults is an important task for the future. We review the evidence pertaining to four neurocognitive systems: threat processing, reward processing, emotion regulation and executive control. For each domain we provide a brief description of the system and its neurocognitive basis, and evidence its role in the context of psychiatric disorder. This is followed by a review of those studies that have investigated the functioning of each system in children and adolescents with histories of maltreatment. In Table 1 we provide an overview of all of the studies reviewed in the current paper, including details regarding age, sample size, nature of maltreatment experience as well as a brief summary of the neurocognitive findings. Following the main body of the review we consider limitations in the extant literature and consider the clinical implications of this research.

**Table 1:** Functional magnetic resonance brain imaging studies investigating the association between early adversity (EA: either institutionalization or maltreatment) and alterations across four neurocognitive domains in children and adolescents

Study	Mean age	Sample size	Task	Assessment	Maltreatment subtype	Compared to controls MT presented
Threat processing						
Maheu et al. (2010)	13.5	11AE/19NAE	FP	IR/OR	ED	Higher AMY and anterior hippocampus activation to fearful and angry faces
McCrory et al. (2011)	12.5	20AE/23NAE	FP	IR	PA/SA/N/EA/DV	Higher AMY and AI activation to angry faces
Tottenham et al. (2011)	10.1	22AE/22NAE	FP	IR	ED	Higher AMY activation to fearful faces
White et al. (2012)	13.5	139#	FP	SR	N	Higher AMY activation in carrier of ‘riskier’ FKBP5 polymorphism
McCrory et al. (2013)	12.5	18AE/23NAE	sFP	IR	PA/SA/N/EA/DV	Higher AMY activation to angry faces and happy faces
Reward processing						
Mehta et al. (2010)	16	12AE/11NAE	OC	IR	ED	Lower BG (NAcc) activation during reward anticipation
Goff et al. (2013)	9.8	38AE/31NAE	PV	IR	ED	Lower BG (NAcc) activation to positive stimuli
Hanson et al. (2015)	13.7	106#	OC	SR	N	Lower BG (NAcc) activation during reward feedback
Dennison et al. (2016)	16.9	21AE/38NAE	PV	SR	PA/SA	Higher BG (NAcc, putamen) to positive stimuli. Higher BG (pallidum and putamen) activation to positive stimuli cross‐sectionally, predicted lower depression symptoms longitudinally
Gerin et al. (in press)	13.1	18AE/19NAE	OC	IR	PA/SA/N/EA/DV	Lower BG (caudate and pallidum), OFC, Insula and hippocampus activation during reward anticipation
Emotion regulation						
Gee et al. (2013)	12.1	41AE/48NAE	FP	IR	ED	Reduced AMY‐mPFC connectivity in younger, but not older, children during affect processing
Marusak et al. (2015)	12	14AE/16NAE	EC	SR/OR	PA/N/DV	Increased AMY‐ACC connectivity and higher dlPFC activation during emotional conflict
Puetz et al. (2014)	10.6	25AE/26NAE	PS	IR/OR	N/PA/DV	Reduced dACC‐dlPFC connectivity and lower dACC and dlPFC activation during social rejection
Lee et al. (2015)	16.12	31#	FP	SR	EA	Reduced AMY‐ACC connectivity during implicit affect processing
McLaughlin et al. (2015)	16.6	21AE/21NAE	EER	SR/OR	PA/SA	Higher dlPFC, mPFC and dACC activation during effortful attempt to decrease emotional response to negative stimuli.
Elsey et al. (2015)	15.4	31AE/33NAE	PI	SR	PA/SA/N/EA	Higher mPFC, lPFC, dACC, PCC and insula activation during personalized stress cues.
Puetz et al. (2016)	12.6	21AE/19NAE	EC/SR	IR	PA/SA/N/EA/DV	Lower vlPFC, insula, AMY and STS activation during emotional conflict for rejection‐themed words
Executive control						
Mueller et al. (2010)	13.5	12AE/21NAE	SS	IR	ED	Higher dACC, vlPFC, BG and insula activation during error monitoring and cognitive control functions (e.g. inhibiting, shifting)
Lim et al. (2015)	17	22AE/27NAE/17PSYCH	SS	IR/OR/SR	PA/EA/N	Higher dACC/MCC and dorsomedial frontal regions activation during error monitoring

Sample: AE, Adversity Exposed; NAE, Nonadversity Exposed; PSYCH, Mixed Psychiatric Comparison Group; #, Maltreatment measured as a continuous variable.

Task: EC, Emotional Conflict Task; EER, Explicit Emotion Regulation; FP, Face Processing; OC, Operant Conditioning Task; PI, Personalized Imagery; PS, Psychological/Psychosocial Stress; PV, Passive Viewing of Emotional Stimuli; sFP, Subliminal Face Processing.

Maltreatment Assessment: IR, Institutional Record; OR, Other Report; SR, Self‐Report.

Maltreatment Subtype: DV, Domestic Violence; EA, Emotional Abuse; ED, Early Deprivation/Institutionalization; N, Neglect; PA, Physical Abuse; SA, Sexual Abuse.

Brain Regions: ACC, Anterior Cingulate Cortex; AI, Anterior Insula; AMY, Amygdala; BG, Basal Ganglia; dACC, Dorsal Anterior Cingulate Cortex; dlPFC, Dorso‐Lateral Prefrontal Cortex; lPFC, Lateral Prefrontal Cortex; NAcc, Nucleus Accumbens; MCC, Midcingulate Cortex; mPFC, Medial Prefrontal Cortex; PFC, Prefrontal Cortex; PCC, Posterior Cingulate Cortex; STS, Superior Temporal Sulcus.

### Threat processing

The ability to detect and respond to aversive and potentially dangerous stimuli is a necessary condition for survival. Human and animal studies demonstrate that significant neurobiological and cognitive resources are dedicated to, and prioritized for, threat detection and response (LeDoux, 2000; Öhman, 2009). The neural system underlying threat processing is shared across most organisms and often operates outside conscious awareness and beyond explicit and effortful control (Öhman, Carlsson, Lundqvist, & Ingvar, 2007). Evidence from human and animal studies suggests that the amygdala, a subcortical medio‐temporal brain structure, plays a critical role in the detection of salient stimuli, and in particular stimuli associated with danger (Phelps & LeDoux, 2005). However, the amygdala does not operate in isolation, but is integral to a wider network: a subcortical pathway that includes the thalamus, the pulvinar nucleus and the superior colliculus has been implicated in amygdala activation (Öhman et al., 2007), while amygdala outputs are mediated by projections to other subcortical structures, such as the hypothalamus, the bed nuclei of stria terminalis and the striatum. The amygdala is also mutually connected with other neighbouring subcortical temporal regions involved in fear conditioning, such as the hippocampus, and with cortical areas involved in regulatory responses and salience detection, including the anterior insula, the dorsal anterior cingulate cortex (dACC) and ventromedial prefrontal regions (Shin & Liberzon, 2010).

#### Why is threat processing important in the study of psychopathology?

The successful navigation of an unpredictable environment depends on our ability to accurately and efficiently detect and respond to threat; as such, it is reasonable to assume that alterations in this system may potentially place an individual at a greater risk of developing maladaptive behaviours. Anxiety disorders, for example, have been associated with both patterns of vigilance (even in environments that are benign) and avoidance (when threat is present and the typical response is to allocate attention to the potential danger) (Shechner et al., 2012; Wald et al., 2013). Inappropriate hypervigilance may reduce the resources available for other important functions and reduce exploratory behaviour (Rogosch, Dackis, & Cicchetti, 2011), while active avoidance of threat may reflect a maladaptive avoidant coping response. Both patterns of processing may be evident in the same individual, depending on the context. Altered amygdala and anterior insula activation have been implicated in several disorders, including posttraumatic stress disorder (PTSD), anxiety and mood disorders (Etkin & Wager, 2007; Kerestes, Davey, Stephanou, Whittle, & Harrison, 2014; Patel, Spreng, Shin, & Girard, 2012), conduct problems (Viding et al., 2012) and drug addiction (Sripada, Angstadt, McNamara, King, & Phan, 2011).

There is preliminary evidence suggesting that hyperresponsiveness to threatening stimuli may predict the likelihood of future symptomatology. Longitudinal studies of healthy adults and adolescents have shown that amygdala activity levels measured *prior* to stressor exposure (such as exposure to stressful life events, deployment in a war zone or witnessing a terrorist attack) can predict the emergence of *later* psychiatric symptoms, poststressor (Admon, Milad, & Hendler, 2013; Admon et al., 2009; Swartz, Knodt, Radtke, & Hariri, 2015). In light of the theory of latent vulnerability one might hypothesize that at least some of those individuals with higher baseline levels of amygdala activation prior to trauma exposure were the same individuals who had experienced elevated rates of childhood maltreatment; of course others may represent individuals who have genetic vulnerability or those who are doubly unfortunate to have both genetic vulnerability and experience of adversity.

#### Functional neuroimaging studies of threat processing in children and adolescents exposed to early deprivation or maltreatment

Studies using a range of behavioural and electrophysiological testing paradigms with maltreated children and adolescents suggest that various forms of early adversity are associated with long‐term impairments in the threat‐ and fear‐processing systems. These changes are detectable as early as 15 months, and include preferential attention to threatening information, heightened neural response to negative stimuli and enhanced perceptual ability for cues associated with danger, such as angry faces (Curtis & Cicchetti, 2013; Pollak & Sinha, 2002; Pollak & Tolley‐Schell, 2003; Pollak, Vardi, Putzer Bechner, & Curtin, 2005). Animal data also point to the existence of a causal link between early adverse experiences (such as reduced maternal care) and long‐lasting neurophysiological changes in brain regions, such as the amygdala, striatum and hippocampus, involved in threat detection and stress responses (Caldji, Diorio, & Meaney, 2003; Caldji et al., 1998; Meaney, 2001).

In recent years, these neurophysiological and behavioural findings have been complemented by a series of fMRI studies. Two early studies focussed on children who had experienced institutional neglect (i.e. early care in international orphanages), a particularly severe form of early adversity that can include profound deprivation. Maheu et al. (2010) recruited a small sample of children (*N* = 11), the majority of whom had experienced orphanage care outside the United States. One notable feature of this early study, that set a benchmark for future studies, was the fact that the comparison group of nondeprived children (*N* = 19) were matched on IQ, age, pubertal status and socioeconomic status (SES), thus reducing the range of potential confounds when interpreting any observed group differences. Greater activation in subcortical regions including the amygdala and hippocampus was observed in the deprived sample during threat processing in a paradigm using emotional faces, even when two participants who met criteria for a psychiatric diagnosis were removed. Amygdala response was negatively correlated with time spent in an adoptive family, suggesting that level of amygdala responsiveness to threat was calibrated in a dose‐dependent manner tracking the level of adversity exposure. Using a face‐processing paradigm in a much larger sample, Tottenham et al. (2011) similarly reported higher amygdala response to threatening facial cues in children exposed to early institutional neglect (*N* = 22) compared to an equal number of never‐institutionalized children. These studies demonstrated the potential for severe – arguably species atypical – early adverse environments to shape neural responses to threat cues.

At the same time, our research group were investigating threat processing in children recruited from social services departments in the United Kingdom, all of who had been exposed to maltreatment in community settings. The majority of these children, like most children referred to social services, had histories of polyvictimization (exposure to more than one form of abuse or neglect). We also recruited control participants who did not differ in relation to chronological age, socioeconomic status, IQ and pubertal stage. In our first study, the processing of facial expressions (angry, sad and neutral) was incidental to the gender decision task the children were asked to complete (McCrory et al., 2011). The group exposed to maltreatment (*N* = 20) showed increased activation in the amygdala and anterior insula when processing angry relative to neutral faces. Like the amygdala, the anterior insula has been implicated in the detection of salient information, as well as playing a role in the integration of bodily sensation, including pain anticipation (Wiech et al., 2010). In this study, we found a modest dose‐dependent neural response in the insula with the degree of violence exposure at home, again pointing to a pattern of neural calibration commensurate with the level of exposure to early adversity. In a second study, we sought to investigate whether altered threat processing was evident in children exposed to maltreatment even at a preconscious stage of awareness, using a dot probe paradigm (McCrory et al., 2013). Facial cues were presented preattentively (i.e. for 17 milliseconds) and backward masked, such that children had no conscious awareness of having viewed faces let alone discriminate their emotional valence (McCrory et al., 2013). As hypothesized, the group of children exposed to maltreatment (*N* = 18), compared to a matched sample of nonmaltreated children (*N* = 23), showed greater neural response in the amygdala to angry relative to neutral faces. Again, amygdala reactivity to threat cues in the maltreated group correlated with indices of maltreatment exposure, notably neglect and emotional abuse, the two most common forms of abuse characterizing the sample. These findings suggested that altered neural response to threat cues in maltreated children is not the result of conscious regulatory control.

In a study with a large sample of typically developing adolescents (*N* = 139), White et al. (2012) explored the possibility that individual differences in amygdala threat reactivity associated with the degree of maltreatment experience – specifically emotional neglect – may in part be accounted for by the presence of genetic differences. In particular, variations of the human gene that codes for the FK506‐binding protein 5 (FKBP5) was investigated. This gene has been previously associated with the emergence of stress‐related psychiatric symptoms. As predicted, emotional neglect was associated with higher amygdala reactivity, but *only* in those adolescents carrying the ‘riskier’ genetic polymorphisms. This resonates with epidemiological studies suggesting that the link between maltreatment and future psychopathology is moderated by genetic variability that can confer increased vulnerability or resilience (Caspi & Moffitt, 2006). One limitation of this study is that the majority of participants’ experience of emotional neglect fell within the normative range, and would not be classified as maltreatment (White et al., 2012). As such, it is not possible to generalize on the basis of these findings to individuals who may have experienced adversity of sufficient severity to warrant professional attention. Future studies, with samples of children and adolescents with documented (and more severe) experiences of emotional neglect, as well as other forms of abuse, would be helpful to further explore the nature of such gene–environment interactions.

Additional evidence for the association between abuse and neglect and the neural responsiveness of the threat‐processing system comes from recent studies of both previously institutionalized children (Gee et al., 2013) and individuals who experienced maltreatment in a community setting (Lee et al., 2015; Marusak, Martin, Etkin, & Thomason, 2015; McLaughlin, Peverill, Gold, Alves, & Sheridan, 2015; Puetz et al., 2016). As these studies primarily focussed on investigating neural connectivity across brain regions during emotion regulation, we consider them in a separate section below. However, it is pertinent to note that in analyses of focal activation, these studies also found that institutionalization and maltreatment experience were associated with altered amygdala response to negative and threatening social cues. In contrast to the other studies described here, Puetz et al. (2016) found that a pattern of *reduced* activity in the amygdala and associated regions (including the insula, ventrolateral prefrontal cortex and orbitofrontal cortex) was associated with maltreatment experience; a group of children exposed to maltreatment (*N* = 21) were compared to 19 matched controls during a Stroop task in which children were required to name the colour of neutral words and socially threatening words associated with rejection. This pattern of *hypoactivation* of the amygdala to social threat cues (and associated regions) has also been observed in patients with PTSD and may reflect a tendency towards threat avoidance related to dissociation symptomatology and an avoidant coping style.

#### Summary

There is a substantial body of evidence suggesting that exposure to early adversity, including institutionalization and maltreatment in a community setting, alters the neural reactivity of the threat system, even in ‘healthy’ children/adolescents who are not presenting with a psychiatric disorder. The degree of reactivity appears to partly relate to the severity of early adversity (Maheu et al., 2010; McCrory et al., 2011, 2013; White et al., 2012) and possibly to genetic differences (White et al., 2012). This pattern of findings is consistent with the view that altered threat reactivity, as indexed by neural response of the amygdala (and related structures) to biological threat stimuli, represents one candidate neurocognitive system conferring latent vulnerability in children exposed to early adversity. As noted earlier, patterns of hyper‐ and hyporeactivity of the amygdala and anterior insula to threat‐related cues have been associated with the clinical presentation of a number of disorders, including anxiety. A recent meta‐analysis combining data from 20 child and adult studies, found that maltreatment experience was reliably associated with increased bilateral amygdala activation to emotional faces, as well as hyperactivation of the parahippocampal gyrus and insula (Hein & Monk, 2017). Longitudinal studies are still needed to establish whether altered threat processing in maltreated samples predicts psychiatric outcome.

When trying to make sense of these findings from studies of deprivation and maltreatment, it can be useful to consider the wider field of research investigating threat detection brain circuitry. For example, threat reactivity before and after combat exposure, a very different kind of environmental danger, has been associated with increased reactivity of the amygdala and anterior insula (e.g. van Wingen, Geuze, Vermetten, & Fernández, 2011) and normalizes 18 months following return from combat (van Wingen et al., 2012). In light of this, we have argued that the pattern of neural response in maltreated children most likely reflects a pattern of *adaptation* to environmental threat rather than a form of ‘damage’. Although such changes may be adaptive in the short term, the theory of latent vulnerability contends that in the long term they contribute to an increased risk of psychopathology.

Future studies are required to establish whether a pattern of heightened neural responsiveness to threat is associated primarily with deprivation (the absence of expected environmental inputs in cognitive and social domains) or exposure to threatening environments, characterized by sexual, physical or emotional abuse (Sheridan & McLaughlin, 2014). The extant data are unable to properly discriminate between these possibilities, given the high cooccurrence of both forms of adversity in existing samples. However, White et al. (2012) note that in their sample it is emotional neglect that is primarily associated with elevated amygdala reactivity. One might speculate that for emotionally neglected individuals, vigilance to environmental danger becomes more necessary in the absence of adequate caregiver monitoring. Arguably then, emotional neglect is a form of deprivation (an absence of expected social inputs) that requires vigilance as an adaptive response in order to maintain physical safety. Conversely, it would be equally plausible to argue that *direct* exposure to environmental danger – events that can harm an individual – is what serves to attune the threat processing system, leading to neural hyperactivity in threat‐related neural structures (McCrory et al., 2011; Sheridan & McLaughlin, 2014). Larger samples that allow researchers to tease apart the differential effects of different forms of maltreatment, as well as systematically controlling for potential covariates of no interest, will be required to discriminate between these possibilities.

### Reward processing

Reward processing plays a central role in our ability to successfully adapt to the environment by motivating and reinforcing goal‐directed behaviour: we seek out natural rewards and learn which neutral stimuli predict rewards, at both conscious and unconscious levels (Berridge, Robinson, & Aldridge, 2009). Berridge and colleagues have suggested that reward processing can be conceptualized as comprising three main components: ‘liking’, ‘wanting’ and ‘learning’. They suggest that each of these components reflect different psychological processes that in turn map onto dissociable neuroanatomical and neurochemical brain reward systems. To date most studies of psychopathology have focussed on the study of wanting or *incentive salience*, a type of motivation that promotes approach towards and consumption of reward, as well as liking. Experimental tasks have typically used secondary rewards such as money or points, or primary rewards, including images of happy faces. Reward anticipation or ‘wanting’ is indexed in particular by neural response in the ventral striatum, with response to reward‐related cues mediated by dopamine signalling. In contrast, ‘liking’ (reflecting a hedonic response signalling receipt of reward) is believed to be mediated by opioid and endocannabinoid systems (Luking et al., 2016).

#### Why is reward processing important in the study of psychopathology?

Neurological alterations in reward processing, such as reduced activity in the striatum, have been implicated in the pathophysiology of several disorders, including depression (Forbes & Dahl, 2012; Olino et al., 2014; Pizzagalli et al., 2009; Ubl et al., 2015), substance abuse (Balodis & Potenza, 2015; Beck et al., 2009; White et al. 2016) and anxiety (Hartley & Phelps, 2012; White et al. 2017). A recent longitudinal fMRI study with a large community sample of adolescents (*n* = 1576) has reported that blunted striatal response during the anticipation of rewards predicts future clinical status and anhedonia in a dose‐dependent fashion (Stringaris et al., 2015). Importantly, this study found that reduced activation in the ventral striatum predicted the emergence of clinical depression and anhedonia at 2‐year follow‐up, even in previously healthy individuals. These and related findings (Bress, Foti, Kotov, Klein, & Hajcak, 2013; Telzer, Fuligni, Lieberman, & Galván, 2014) suggest that alterations in the reward network are not only implicated in the pathophysiology of depression but may also represent a marker of vulnerability. Animal studies have also reported decreased neural signalling in the striatum (especially in dopaminergic activity) and the emergence of depression‐like behaviours, such as reduced motivation towards reward‐predicting cues (i.e. anhedonia), as well as increased neural (and behavioural) sensitivity towards the addictive properties of drugs (Andersen & Teicher, 2009; Hall, Wilkinson, Humby, & Robbins, 1999; Kosten, Zhang, & Kehoe, 2005; Matthews & Robbins, 2003; Pryce, Dettling, Spengler, Schnell, & Feldon, 2004). Such neural alterations in reward processing can be caused by stress exposure, especially in infancy (Meaney, Brake, & Gratton, 2002; Pizzagalli, 2014).

#### Functional neuroimaging studies of reward processing in children and adolescents exposed to early deprivation or maltreatment

In an early study investigating the association between institutionalization and alterations in reward processing, Mehta and colleagues reported blunted neural response in the ventral striatum during the anticipation of monetary rewards in a small group of Romanian adoptees (*N* = 12; Mehta et al., 2010). A study using ‘social reward’ cues (happy faces) with children and adolescents who had experienced international adoption (*N* = 38) compared to a comparison group of peers (*N* = 31) reported a similar pattern, with reduced response in striatal regions, specifically the nucleus accumbens (NAcc) (Goff et al., 2013). Furthermore, lower NAcc reactivity was found to be correlated with higher depression scores (Goff et al., 2013). However, in both studies the institutionalized and comparison groups differed across one or more domains, including social, psychiatric and cognitive functioning making it difficult to confidently attribute the observed differences in neural reactivity to early experience rather than concurrent difficulties.

More recently, three studies have investigated reward processing in individuals who experienced maltreatment in a community setting. In a study of healthy adolescents (*N* = 106) using a monetary incentive delay task, Hanson et al. (2015) found that, independently of clinical status, self‐reported emotional neglect across two time points, was associated with blunted striatal response during reward processing. In turn, decreases in reward‐related ventral striatal activity were associated with greater depressive symptomatology and partially mediated the association between emotional neglect and subsequent depressive symptomatology. A dose‐dependent effect of emotional neglect on brain response was reported, again consistent with the view that level of maltreatment experience appeared to calibrate the level of neural responsiveness. However, these findings were based on a sample where levels of maltreatment experience were assessed in a continuous fashion across the group. Inspection of the means indicates that all forms of maltreatment experience, including emotional neglect, were generally in the minimal to low range. As such, it was not possible to conclude that the findings from this study would be generalizable to definitions of ‘maltreatment’ in a professional context. However, recent findings from our own group using a computational model‐based fMRI paradigm in order to investigate reward anticipation were consistent with the data from Hanson and colleague's study. Neural activation in a group of children and adolescents exposed to independently documented maltreatment experience (*N* = 18) was contrasted with that in a group of carefully matched peers (*N* = 19). Maltreatment experience was found to be associated with a pattern of reduced activation to reward cues in the striatum, and in other regions implicated in outcome representation, including the orbitofrontal cortex and insula (Gerin et al., in press).

In contrast, Dennison and colleagues report *greater* BOLD response during passive viewing of positive relative to neutral social stimuli in the left nucleus accumbens and left putamen in a group of older adolescents (*N* = 21) who had experienced at least moderate physical and/or sexual abuse (ascertained via self‐report and interview) compared with a control group (*N* = 38) matched on age and IQ (Dennison et al., 2016). This is somewhat at odds with what might have been predicted on the basis of the findings by Hanson et al. (2015) and the depression literature. However, maltreatment was associated with depression symptoms only among youth with low reactivity to reward both behaviourally (in a monetary incentive delay task) and neurally (when passively viewing positive images), specifically in the left pallidum. Prospectively (at 2‐year follow‐up), maltreatment predicted increases in depression symptoms over time *only* for adolescents with low, but not high, activation of the left putamen to positive images. The authors suggest that greater reactivity to positive and rewarding environmental cues may be associated with resilience to depression among adolescents who have experienced maltreatment. This study benefits from a well‐characterized group of adolescents who meet criteria for maltreatment and who are carefully matched with nonmaltreated peers. However, dividing the 21 adolescents in the maltreatment sample into ‘low’ and ‘high’ reactivity to reward groups means that these findings are based on a relatively small sample size and require replication. Nonetheless, this study is important in its refreshing focus on potential markers of *resilience* to future mental health symptoms.

#### Summary

To date, functional neuroimaging studies of adolescents indicate that childhood maltreatment leads to alterations in reward processing systems in subcortical reward‐related areas, such as the striatum. Teicher and Samson (2016) have proposed that heightened threat reactivity alongside blunted anticipatory responses to reward (Hanson et al., 2015; Gerin et al., in press), may reflect an adaptive calibration towards an avoidant response during approach‐avoidance conflict situations, increasing likelihood of survival in an environment characterized by danger. However, they note that such altered responsiveness may confer increased risk of depression/anhedonia (Pizzagalli et al., 2009; Wacker, Dillon, & Pizzagalli, 2009), anxiety (Etkin et al., 2004; Redlich et al., 2015) and addiction (Balodis & Potenza, 2015; Corral‐Frías et al., 2015).

More generally, altered reward processing might be understood in the context of a child growing up in an environment where sources of reward are, in reality, unpredictable and scarce: reduced anticipation of reward may simply be calibrated in line with learnt contingencies. In addition, such calibration may reduce the likelihood of experiencing repeated episodes of disappointment, and as such would represent an adaptation that helps the child regulate their internal state within a deprived environment. However, the blunting of anticipatory response would incur a cost, hampering exploratory behaviour in novel environments outside the home, reducing the likelihood of identifying sources of reward even when these are in fact available. It is not difficult to imagine how this would set up a negative reinforcing cycle where a child is less expectant of rewards and less motivated to exert the effort required to attain them. This will have profound implications for how a child both experiences and shapes their environment, as well as how they develop an internalized sense of agency and mastery. Dennison et al. (2016) highlight the potential role for an elevated responsiveness to (rather than anticipation of) reward cues in conferring resilience to depression. Why might some children who have experienced maltreatment show such heightened responsiveness? We suggest that the ability to *recalibrate reward systems* and learn new contingencies in more normative environmental contexts may be a key aspect of resilience (van Wingen et al., 2012). We discuss this further in the final section of the review.

### Emotion regulation

There is no standard definition for what emotions are and, as a consequence, of what emotion regulation entails (Cole, Martin, & Dennis, 2004). However, most researchers generally agree on an evolutionary view of emotions as biological predispositions that have evolved to help us appraise environmental stimuli and prepare us for action. In other words, emotions are viewed as a complex neurophysiological phenomenon encompassing both the evaluation of the environment as well as changes in our motivational state and behaviour. In turn, emotion regulation has been conceptualized as the ability to produce changes in an activated emotion, including the modification of its valence (i.e. positive or negative), intensity or duration (Cole et al., 2004; Eisenberg & Spinrad, 2004; Ochsner et al., 2004). Affect or emotion regulation is regarded as a dynamic and multifaceted process which can operate within or outside our conscious awareness (e.g. Williams, Bargh, Nocera, & Gray, 2009) and it comprises various mechanisms and strategies, including (for example) cognitive reappraisal, suppression and attention modulation (Koenigsberg et al., 2010; Ochsner, Silvers, & Buhle, 2012).

Neuroimaging and lesion studies have identified a functionally and structurally interconnected circuit involved in emotion regulation. In particular, subcortical/limbic regions, involved in the evaluation of threat, reward and internal physiological states (such as the striatum, amygdala and insula) have been found to be strongly interconnected with frontal association cortices (such as the anterior cingulate cortex (ACC) and also medial and lateral prefrontal regions (mPFC and lPFC)) (Ochsner et al., 2012). These frontal brain areas are involved in integrating information from various sensory modalities and have been implicated in a number of processes involved in successful emotion regulation, including assessing one's own and others’ mental states, monitoring conflicting information, inhibiting and selecting behavioural responses and also in attributing context‐dependent value to stimuli. Traditionally, prefrontal regions, such as the ACC, have been understood to exert a top‐down inhibitory effect over subcortical brain structures, such as the amygdala (Hariri, Mattay, Tessitore, Fera, & Weinberger, 2003; Kim, Somerville, Johnstone, Alexander, & Whalen, 2003).

#### Why is affect regulation important in the study of psychopathology?

Difficulties in interpreting and regulating emotions are common features of many psychiatric disorders, including those related to childhood maltreatment (e.g. anxiety, depression, conduct disorder and substance abuse disorders) (Aldao, Nolen‐Hoeksema, & Schweizer, 2010; Mennin, Holaway, Fresco, Moore, & Heimberg, 2007). Deficits in emotion regulation are not only associated with psychopathology, but growing evidence suggests that alteration in emotion processing may represent a risk factor for the emergence of future psychiatric conditions and difficulties in social functioning (e.g. Keenan, 2006). Furthermore, findings from cross‐sectional and longitudinal studies with maltreated children suggest that differences in emotion regulation abilities may represent a risk (or resiliency) factor for the development of future psychopathology (Kim & Cicchetti, 2010; Shields & Cicchetti, 2001). For example, in a longitudinal study of 171 children who had experienced maltreatment and 151 control children, maltreatment experience was associated with high emotion lability/negativity at an age of 7 years that contributed to poor emotion regulation (age 8), which in turn was predictive of increases in internalizing symptomatology a year later (Kim‐Spoon, Cicchetti, & Rogosch, 2013). Such longitudinal work suggests that poor emotion regulation is involved in the development of internalizing symptomatology, in particular anxiety.

#### Functional neuroimaging studies of emotion regulation in children and adolescents exposed to early deprivation or maltreatment

To date, seven studies with children and adolescents with histories of maltreatment have investigated the brain circuitry involved in emotion regulation (Elsey et al., 2015; Gee et al., 2013; Lee et al., 2015; Marusak et al., 2015; McLaughlin et al., 2015; Puetz et al., 2014, 2016). The findings of these studies largely converge, indicating functional alteration in a group of brain regions implicated in emotion regulation. Specifically, the findings suggest atypical focal brain activity in regulatory regions such as the ventral ACC (vACC) and the lPFC as well as alterations in functional connectivity between frontal and subcortical brain regions, including the amygdala–vACC circuitry. However, despite such general agreement, the direction of neural responses and pattern of connectivity (i.e. whether increased or decreased) has varied considerably across studies.

Three studies have investigated the *functional connectivity* between the amygdala and frontal regulatory regions in the context of early adversity. The first such study in children recruited a sample exposed to early institutional neglect. Gee et al. (2013) examined the functional coupling between the amygdala and mPFC during the viewing of emotional faces in 41 children and adolescents who had previously been institutionalized and an age‐matched control group (*N* = 48). Differences only emerged in the subset of younger children (not the adolescents) within the institutionalized sample: they showed a pattern of more negative connectivity similar to that seen in the older adolescents across both groups. The authors suggest that this pattern could be understood as an acceleration of amygdala–mPFC development that could reflect an ontogenetic adaptation in response to early adversity. Using a similar face‐processing paradigm, Lee et al. (2015), in a group of typically developing adolescents (*N* = 31), found that more negative functional connectivity between amygdala activity and the rostral ACC was associated with increased levels of self‐reported verbal abuse and levels of current depression symptoms. A third study examined vACC–amygdala connectivity during an automatic emotion regulation task in a group of trauma‐exposed children (*N* = 14; predominantly maltreatment exposure) and a group of control children matched for age, IQ, pubertal status and a measure of SES. The group of trauma‐exposed children did not show a typical pattern of negative connectivity between the amygdala and the vACC unlike their peers (Marusak et al., 2015). While each of these findings point to an association between early adversity and altered amygdala–PFC connectivity during emotion regulation, the direction of findings are notably discordant. This is perhaps not surprising given the range of participants recruited (previously institutionalized, typically developing, trauma exposed), the range in ages of participants and the use of different types of tasks (simple affect processing tasks (Lee et al., 2015; Gee et al., 2013) versus more complex tasks of emotional conflict processing (Marusak et al., 2015). It is likely that a complex pattern of altered connectivity characterizes individuals exposed to early adversity that is related to developmental stage, the nature of adversity experienced and the specific computational demands of any given emotional processing task. This remains to be further investigated in future studies.

Five studies have investigated brain *activity* in children exposed to maltreatment during a number of paradigms requiring emotion regulation. The main differences in brain responses between the maltreated and nonmaltreated group of children, across all studies, clustered around similar brain regions implicated in the studies of brain connectivity just discussed, including the ventral and dorsal ACC and the lPFC (Elsey et al., 2015; Marusak et al., 2015; McLaughlin et al., 2015; Puetz et al., 2014, 2016). Three studies found an overall pattern of *increased* activity in the dorsolateral PFC and also in the dorsal and ventral ACC (Elsey et al., 2015; Marusak et al., 2015; McLaughlin et al., 2015). Conversely, two studies by Puetz and colleagues (Puetz et al., 2014, 2016) found that maltreatment was associated with *reduced* activity in these brain regions during the processing of information related to social rejection. There are many differences across the paradigms used in these studies that make interpretation of such contrasting findings challenging. However, it is arguable that while atypical emotion regulatory responses characterize children exposed to maltreatment the *direction* of the response may partly depend on the degree to which the processing of any aversive stimuli is mandated by the context or avoidable by some form of distraction. For example, when participants are required to explicitly modulate their emotional responses to visually presented stimuli (McLaughlin et al., 2015) or listen to individualized scripts of stressful experiences (Elsey et al., 2015), increased activation in frontal regulatory regions is observed potentially reflecting greater effort. In contrast, when participants have latitude to process aversive stimuli more incidentally such as during the colour naming of rejection‐related words (Puetz et al., 2016) or the processing of rejection during a simulated game (Puetz et al., 2014), decreased activation of the same regions is observed, potentially reflecting greater avoidance. Indeed it may be the case that automatic avoidant coping responses to negative cues, which may be a strategy to reduce negative affect in the short term, could over time compromise the development of explicit emotion regulation skills. Consistent with this hypothesis, behavioural data indicate that maltreated individuals may direct attention away from stimuli that elicit negative and discomforting affect (Kelly et al., 2015; Pine et al., 2005) and suggest that the degree of threat avoidance partly mediates the relationship between maltreatment and level of emotional reactivity (Kelly et al., 2015).

#### Summary

Neuroimaging studies with children exposed to maltreatment indicate functional alteration in a group of brain regions and networks traditionally associated with emotion regulation/‘hot’ executive control. Specifically, atypical *connectivity* and focal *activity* has been found in fronto‐limbic neural circuits, including the ventral ACC (vACC) and the amygdala, and also in lateral frontal regions. Anomalies in other regions crucial for the cognitive modulation of affect (such as the dorsolateral prefrontal cortex) and for the automatic regulation of the hormonal stress responses (such as the hippocampus) have also been implicated. However, the direction and pattern of functional alterations has varied across studies. Differences in the direction of effects across the functional connectivity studies may in part relate to variation in the ages and forms of adverse experiences characterizing each sample. Differences in the direction of effects across the focal activation studies, in contrast, may relate to the paradigm used, task context and the scope within any given paradigm for participants to simply engage in avoidance of negative stimuli; such avoidance may reduce the processing of aversive stimuli in the short term at the expense of the development of effective emotion regulation skills. Future work is needed to test these hypotheses. McLaughlin et al. (2015), in their study of explicit emotion regulation, conclude that the greater engagement of PFC regions in their maltreated sample reflects increased allocation of cognitive resources to effortfully modulate emotional responses. They also highlight the potential importance of cognitive reappraisal strategies in any therapeutic intervention. Such a component could be usefully incorporated in a future longitudinal design that also explicitly assesses whether alteration in regulatory networks during childhood represents a true marker of future psychopathology and thus a marker of latent vulnerability.

### Executive control

#### What is executive control?

Planning, flexible thinking and anticipating outcomes are crucial to accomplish typical day‐to‐day activities as well as to achieve long‐term goals (Snyder, Miyake, & Hankin, 2015) and are generally understood to reflect executive control. According to an influential model, there are three basic cognitive functions underlying executive control: updating, inhibiting and task shifting (Miyake et al., 2000). Updating is closely related to the concept of working memory. It refers to the capacity to manipulate and maintain information in an active state (e.g. sustained attention) and to disregard distracting inputs (e.g. attention control) (Hofmann, Schmeichel, & Baddeley, 2012). Inhibitory control refers to the ability to constrain automatic or dominant behavioural and cognitive responses irrelevant or counterproductive to the achievement of a given goal (Funahashi, 2001). Shifting consists of the ability to switch back and forth between different tasks, mental states and concepts. These functions interact together to bring about effective decision‐making and adaptive behaviours, including self‐regulation and the ability to monitor performance and detect errors (i.e. error processing). These ‘cold’ executive control functions rely more heavily on lateral and dorsomedial frontal regions, the basal ganglia, the thalamus and posterior parietal regions (Rubia, 2011).

#### Why is executive control important in the study of psychopathology?

Impaired executive control is associated with emotion regulation difficulties, rumination and reduced social skills, which are all predictors of psychopathology (Snyder, Kaiser, Warren, & Heller, 2015; Snyder, Miyake et al., 2015). There is a large body of cross‐sectional evidence linking behavioural and neurological measures of executive control with several psychiatric disorders, ranging from depression, anxiety, ADHD, conduct problems, OCD, PTSD and psychosis (Cortese et al., 2012; Eysenck, Derakshan, Santos, & Calvo, 2007; Holmes et al., 2005; Morgan & Lilienfeld, 2000; Rubia, 2011; Snyder, 2013; Snyder, Kaiser et al., 2015; Snyder, Miyake et al., 2015; Willcutt, Doyle, Nigg, Faraone, & Pennington, 2005). Moreover, evidence from longitudinal studies suggest that, independently of baseline diagnostic status or symptoms level, executive control functioning predicts future symptoms of PTSD (Parslow & Jorm, 2007), depression and anxiety (Evans, Kouros, Samanez‐Larkin, & Garber, 2015; Han et al., 2015), ADHD (Campbell & von Stauffenberg, 2009) and psychosis (Cannon et al., 2006).

#### Functional neuroimaging studies of executive control in children and adolescents exposed to maltreatment

Two studies with maltreated children have employed similar versions of the stop‐signal paradigm in order to investigate executive control. Mueller et al. (2010), recruited a group of 12 adopted children and adolescents who had been exposed to early adversity, including institutional neglect and a group of IQ‐ and SES‐matched controls (*N* = 21). Increased activity in brain regions associated with executive control (potentially reflecting decreased neural efficiency/greater effort) was found in the group exposed to early adversity, including in the dorsal ACC (dACC), and lateral frontal regions during error processing, cognitive shifting and inhibitory responses. This is consistent with the neuroimaging literature of various psychiatric disorders, including ADHD (Cortese et al., 2012), anxiety (Basten, Stelzel, & Fiebach, 2011), psychosis (Callicott et al., 2000) and depression (Harvey et al., 2005). In a subsequent study, Lim et al. (2015) also used a stop‐signal task that was individually adjusted in order to elicit 50% of inhibitory errors in a group of older adolescents exposed to maltreatment (*N* = 22), an age and SES‐matched healthy control group (*N* = 27) and psychiatric comparison group (*N* = 17). As hypothesized, during failed inhibitory responses the adolescents exposed to maltreatment showed increased activity in regions traditionally associated with error processing and inhibition compared with the control group. In particular, increased activity was observed in the dorsal dACC, the middle cingulate cortex (MCC) and in lateral frontal regions, including the supplementary motor area. Differences in the supplementary motor area were evident in comparison with the psychiatric comparison group, suggesting that alterations in the brain network involved in executive control are related to maltreatment exposure and are not just an epiphenomenon of concurrent psychopathology.

#### Summary

Rather surprisingly and despite a relatively large behavioural literature, only two studies using fMRI have investigated the neural correlates of executive control in children and adolescents who have been exposed to institutionalization or maltreatment. Findings from these studies indicate increased activity during error monitoring and inhibition in medial and lateral frontal regions, such as the dACC and frontal motor regions consistent with altered executive control following maltreatment experience, which may in turn increase risk of future psychopathology. Such an interpretation should, however, be considered in the light of recent findings from two large longitudinal cohorts (Danese et al., 2016). Danese and colleagues capitalized on multiple assessment time points in these cohorts in order to investigate whether there is a *causal* relationship between childhood victimization and impairments in cognitive functioning. Rather strikingly, the data demonstrated that although individuals with a history of childhood victimization were characterized by deficits in general intelligence and executive control (in line with the extant literature), such deficits were largely explained by cognitive deficits present *prior to* the experience of childhood victimization and by nonspecific effects of childhood socioeconomic disadvantage (Danese et al., 2016). Thus, the neuroimaging findings with respect to executive control reported here need to be viewed with a degree of caution, as they may not reflect alterations in cognitive processes associated with maltreatment per se, but rather reflect prior cognitive vulnerabilities.

## Conclusions: Implications for research

In a relatively short period there has been a welcome proliferation and interest in how early adversity in general, and maltreatment in particular, are associated with alterations in a set of candidate neurocognitive systems that may embed latent vulnerability to future mental health problems. However, a degree of caution is required regarding the level confidence we can have in these findings; to advance the field we need to acknowledge and learn from the limitations that characterize many of our studies to date. While there have been welcome advances in neuroimaging techniques and analytic approaches, there has been less progress in what might be considered more basic aspects of study design. This may stem from the difficulty in defining and measuring maltreatment and recruiting participants who often find it less easy to volunteer for research studies for a variety of reasons; this may partly account for the fact that neuroimaging studies of maltreatment have tended to have relatively small sample sizes. In addition, it has been difficult, based on the extant fMRI studies, to make any claims about sensitive (or critical) periods (i.e. limited periods of time during which the effects of experience on brain and behaviour are particularly potent; Knudsen, 2004). This is because there is great heterogeneity in timing of exposure of abuse among children exposed to maltreatment in community settings. On the other hand, animal models, and human behavioural and electroencephalography (EEG) studies of institutional deprivation (e.g. Levine, Huchton, Wiener, & Rosenfeld, 1991; Zeanah, Gunnar, McCall, Kreppner, & Fox, 2011) have allowed some important inferences to be made regarding sensitive periods in development. In these studies it was possible to identify (or even manipulate) the time of exposure to an adverse environment. More broadly, the functional neuroimaging studies reviewed here involve the cross‐sectional evaluation of neurocognitive function and maltreatment experience, precluding any strong inference regarding causality. Here, we set out five suggestions to improve future studies to enhance confidence in the validity of their findings.

1. *Ensure effective matching*: First, in any study comparing a group of children and adolescents exposed to adversity with a control sample, it is essential that groups are matched at the very least on chronological age, IQ, sex, a measure of SES and in samples including younger adolescents, a measure of puberty. Such matching ensures that any observed differences can be more confidently attributed to the maltreatment experience (rather than, say, poorer cognitive functioning or general deprivation). Matching on psychiatric symptoms is not recommended as such an approach is likely to remove variance of interest given that both internalizing and externalizing symptoms are associated with maltreatment history. However, it is prudent not to conflate maltreatment experience and presenting psychiatric disorder by conducting any comparisons with and without the proportion of any sample meeting clinical criteria (or cutoffs) for a psychiatric disorder, to ascertain whether the presence of clinical disorders are driving any observed differences.
2. *Fully characterize all maltreatment domains*: Many of the studies reviewed here have chosen to provide only partial or incomplete descriptions of their sample in terms of the kinds of maltreatment experiences to which children were exposed. Some have selected their sample on the basis of only one or two maltreatment subtypes (e.g. physical, sexual or emotional abuse or neglect) or failed to fully characterize their sample across maltreatment subtypes. We know that polyvictimization is the normative experience for most children exposed to maltreatment (e.g. Radford, Corral, Bradley, & Fisher, 2013). To select participants on the basis of only one subtype of maltreatment (and disregard or simply ignore the others) means that the complete maltreatment history in the experimental and comparison groups is not fully captured. This unmeasured adversity may be what is in fact driving any observed effects rather that the maltreatment subtype on which the researchers have chosen to focus.
3. *Do not conflate adversity in the normal range with maltreatment exposure:* A number of studies have recruited participants from the typical population and used a self‐report measure of maltreatment or adversity, then implemented correlational or regression‐related designs. This approach can be helpful in examining how a form of adversity across the continuum of experience may be associated with alterations in neurocognitive functioning (e.g. occasional poor supervision at one end to frank neglect at the other; unpleasant verbal comments at one end to derogatory verbal abuse at the other). However, this approach is less helpful in drawing strong inferences regarding the possible impact of maltreatment as judged within a clinical/social care context – that is, an experience reflecting abnormal and excessively harsh caregiving that would warrant professional attention. When a sample is largely comprised of typically developing children, the findings may well tell us a great deal about development following *everyday* levels of adversity but may have more limited relevance when drawing inferences about children or adolescents exposed to *actual* maltreatment. Any conclusions about the impact of maltreatment experience require sufficient data on children who have suffered actual maltreatment within any broader sample and a clear analysis strategy that enables inference on the impact of nonnormative levels of adversity.
4. *Take particular care in measuring emotional abuse using self‐report instruments*. The Childhood Trauma Questionnaire (CTQ) is a self‐report measure commonly used to index levels of physical, sexual and emotional abuse, as well as physical and emotional neglect (Bernstein & Fink, 1998). In one study of over 2000 male adolescents aged 12–14 years using the CTQ, it was found that over half met threshold for ‘severe’ or ‘extreme’ emotional abuse, while the rates of other forms of maltreatment were in line with what might be expected based on clinical experience (Mikaeili, Barahmand, & Abdi, 2013). Inspection of the items for emotional abuse in the CTQ reveal that they relate to common experiences for many children – being called ‘lazy’ or ‘stupid’ by people in one's family (including, for example, siblings) or having family members say ‘hurtful or insulting things to them’. In some contexts such comments may simply be unkind; in other contexts, however, they may be part of a denigrating or humiliating pattern of treatment. Unfortunately the CTQ appears poorly equipped to differentiate between these possibilities. A separate issue arises when trying to measure the relationship between self‐reported emotional abuse and risk of depression. The items pertaining to emotional abuse potentially conflate depressive schemas capturing the expectation (rather than the reality) that others will hurt, abuse, humiliate, cheat, lie, manipulate or take advantage (van Vlierberghe, Braet, Bosmans, Rosseel, & Bögels, 2010) with actual experiences of emotional abuse. Maladaptive schemas have been shown to powerfully predict future depressive symptomatology even when baseline levels of depression have been taken into account (Friedmann, Lumley, & Lerman, 2016). As such, measurement of emotional abuse in particular appears to warrant, where possible, appropriate use of stringent thresholds, structured interviews and/or independent verification. New research focusing on the psychometric properties of the emotional abuse scale of the CTQ, as well as its relation with depressive schemas is also warranted. In addition, recent evidence proceeding from large epidemiological data (Reuben et al., 2016) suggests that there is only a moderate association between retrospective and prospective assessments of early adversity. Furthermore, compared to prospective assessments, retrospective measures of childhood experience were shown to have weaker association with life outcomes that were objectively assessed. This suggests that, when possible, researchers should attempt to use prospective and objective measures of maltreatment, such as institutional records (e.g. from child protection services).
5. *Longitudinal designs:* In almost all of the studies reviewed here, psychopathology was measured concurrently with neurocognitive functioning. Longitudinal prospective studies are required to establish whether alterations in neurocognitive functioning can predict levels of future psychiatric symptomatology; this would provide evidence that they may be mechanistically implicated in the pathogenesis of a given disorder. Without such empirical evidence the theory of Latent Vulnerability remains untested. To date the theory is supported by preliminary evidence from two seminal studies that have measured psychopathology after neurocognitive functioning was assessed, allowing (for the first time) a stronger causal inference to be made (Dennison et al., 2016; Hanson et al., 2015), as well as by neurocognitive evidence from other populations (see McCrory & Viding, 2015). While longitudinal studies of frank maltreatment in particular are challenging, given the frequent nature of placement changes of children who are in the care system, they are essential if we are to identify accurate indices of psychiatric vulnerability and shed light on those mechanisms that may represent important targets for preventative intervention. That said, it is unclear whether the neurocognitive systems investigated to date represent those that are most directly implicated in the pathogenesis of psychiatric disorder. Finally, it is important to be mindful of the limitations of the extant studies, which have been cross‐sectional in nature. Specifically, it is not possible to definitively establish a causal relationship between maltreatment exposure and altered neurocognitive functioning. However, two strands of evidence support such an inference. First, evidence from animal studies indicating altered brain structure and function following adverse early care (e.g. Ichise et al., 2006; Spinelli et al., 2009). Second, the preliminary evidence pointing to a dose‐dependent effect of maltreatment exposure and altered neurocognitive functioning in children and adults (e.g. Dannlowski et al., 2012; McCrory et al., 2013).

## Conclusions: implications for clinical practice

These neuroimaging findings, across neurocognitive domains are consistent with the view that maltreatment experience in childhood may embed latent vulnerability to future poor mental health by altering specific aspects of functioning. Two features of the research findings reviewed above are particularly notable from a clinical perspective. Firstly, that alteration in specific aspects of neurocognitive functioning is evident even in the absence of presenting psychiatric disorder; and secondly, that the patterns of atypical neural functioning associated with maltreatment experience are remarkably similar to those seen in psychiatric disorders associated with maltreatment. As such, these neuroimaging findings are beginning to shape a new understanding of a very old problem – how early experience can have such an enduring impact on mental health many years after maltreatment exposure (e.g. Widom et al., 2007). Neurocognitive alterations may hold functional value for the child in the context of an early neglectful or abusive home environment. But they may also serve to contribute to the pathogenesis of future poor mental health problems, ceasing to be adaptive or beneficial during later stages of development or in more normative environments (McCrory & Viding, 2015). In this review, we have considered evidence from four neurocognitive domains. However, a number of other domains require investigation, including autobiographical memory processing, learning and social affiliation, as well as subdomains within the neurocognitive domains reviewed here.

How do alterations in specific neurocognitive systems impact individuals in ways that make them more vulnerable to future stressors? We suggest that latent vulnerability can be considered to unfold in at least two ways (see Figure 1). First, there may be direct effects on immediate processing of the internal and external world. For example, increased allocation of attention to threat cues may reduce the attentional capacity available to be invested in more normative aspects of social and cognitive development, reducing the degree to which an individual is able to process other potentially helpful cues in their environment (McCrory et al., 2011). Similar direct effects may arise, for example, following increased allocation of resources during overt emotion regulation (McLaughlin et al., 2015), reducing resources for other aspects of functioning. Second, there may be indirect effects that serve – over time – to compromise the development of the social support network around the child or adolescent. For example, altered patterns of threat vigilance and avoidance may increase the risk of conflictual social interactions making it more difficult for the child to build stable friendships that can help buffer the impact of future stressors (Puetz et al., 2014, 2016). Similarly, attenuated reward processing may increase anhedonia, reducing the motivation to engage in novel activities or social interactions that may in turn curtail the development of supportive peer friendships. These latent vulnerability effects, both direct and indirect, will over time reduce the degree of resilience shown by an individual in the face of a future stressor, thereby increasing the probability that a mental health problem will emerge. It may be that a shared goal of preventative interventions will be to augment the child's ability to form secure and functional relationships (Toth, Gravener‐Davis, Guild, & Cicchetti, 2013) but how this is achieved may vary depending on the particular profile of latent vulnerabilities with which a child presents. It is likely that each child may present with a unique profile of strengths and vulnerabilities that will need to be considered in any clinical formulation. The end goal of promoting functional social adaptation would, however, remain the same.

An important outstanding question for clinicians and researchers alike relates to the malleability of neurocognitive systems in ‘recalibrating’ responses to threat and reward cues in line with more normative environmental contingencies. It will be important to investigate the range of possible factors that may be implicated in promoting malleability and change in relation to the functioning of those neurocognitive systems associated with latent vulnerability. One framework that takes a developmental perspective focuses on the child's relationship with a sensitive and warm caregiver who understands the child to be an intentional agent capable of representing their mental states. Such an understanding is thought to be critical for the development of epistemic trust, a necessary building block for using social referents to acquire new knowledge about the world (Fonagy & Allison, 2014). In the case of maltreatment, where the caregiver shows an absence of mentalizing and contingent and marked mirroring, the development of epistemic trust is compromised. This leads to a child mistrusting the information conveyed by others, limits their ability to learn about the cultural and interpersonal world, and developing effective mentalization skills themselves (Bo, Sharp, Fonagy, & Kongerslev, 2015; Fonagy & Allison, 2014). One might hypothesize that young people most able to show recalibration – reconfiguring their responses to environmental and internal threat and reward‐related cues following maltreatment exposure to more benign environments and social interactions – have the greatest levels of epistemic trust. Enhancing such trust (and consequent learning) by typical adults, even in those who are ordinarily capable of sensitive caregiving, may be insufficient to meet the needs of those children with altered social information processing and marked behavioural problems. How to develop an effective set of therapeutic tools to enhance and promote the development of epistemic trust then becomes a challenge, which could be seen as one important factor in promoting a resilient outcome.

Arguably the most important implication of these neuroimaging findings is that they provide both the motivation and the rationale to pursue a much more explicitly preventative psychiatry approach in helping those children exposed to maltreatment *before* they present with a frank psychiatric disorder. Current models of social care and mental health provision are typically organized in such a way that discourages innovation in the field of indicated prevention – where those children presenting with the greatest latent vulnerability might be identified and offered a form of intervention to offset the likelihood of a future mental health problem emerging. At the level of social care the priority is to address child protection and welfare concerns, ensuring that children are safe and if necessary moved into an alternative setting when maltreatment is established. At the level of mental health service provision the priority is to treat children who meet criteria for a psychiatric disorder. There is almost no provision for those children who have experienced maltreatment but who do not present with a manifest psychiatric disorder; indeed, such children have generally not been viewed as the concern of mental health professionals at all despite a compelling evidence documenting the significantly elevated risk of future disorder that characterize these children (Vachon et al., 2015). In such individuals, manifest disorders may not emerge for many years following the maltreatment experience (e.g. Teicher, Samson, Polcari, & Andersen, 2009). Such polarization along social care and mental health lines (and the absence of a substantial role for mental health provision in schools) has meant that there has been little space or incentive for clinicians (or other professionals) to develop models of preventative intervention for children who have experienced maltreatment, but do not immediately meet criteria for a psychiatric disorder. Developing a neurocognitively informed screening tool capable of accurately indexing latent vulnerability is essential if we are to identify those children who are not yet overtly symptomatic but who are at most risk of future psychiatric disorder. More broadly, by establishing a better understanding of the specific neurocognitive mechanisms implicated in the pathogenesis of psychiatric disorder we will be much better placed to develop effective preventative interventions that increase the likelihood of resilient outcomes.

**Key points**

- Relatively recent fMRI research has demonstrated that childhood maltreatment is associated with altered functioning in a range of neurocognitive systems including: threat processing, reward processing, emotion regulation and executive control.
- Such changes are observable even in the absence of psychiatric disorder and in some cases, predict future symptomatology. They are thought, in part, to reflect adaptations to early adverse environments.
- These changes are strikingly consistent with those seen in individuals presenting with psychiatric disorder suggesting such neurocognitive ‘adaptations’ embed latent vulnerability to future psychiatric disorder.
- These findings establish a compelling case to develop a more precise mechanistic understanding of the pathogenesis of psychiatric disorder following maltreatment and the need to invigorate efforts to build a preventative clinical approach.
